# Diffusion Tensor Magnetic Resonance Imaging of the Brain in APP Transgenic Mice: A Cohort Study

**DOI:** 10.1371/journal.pone.0067630

**Published:** 2013-06-28

**Authors:** Hans-Peter Müller, Jan Kassubek, Ina Vernikouskaya, Albert C. Ludolph, Detlef Stiller, Volker Rasche

**Affiliations:** 1 Department of Neurology, University of Ulm, Ulm, Germany; 2 Experimental Cardiovascular Imaging, Core Facility Small Animal MRI, University of Ulm, Ulm, Germany; 3 Target Discovery Research Germany, Boehringer Ingelheim Pharma GmbH & Co. KG, Biberach, Germany; University of Cambridge, United Kingdom

## Abstract

**Introduction:**

Fast *in-vivo* high resolution diffusion tensor imaging (DTI) of the mouse brain has recently been shown to enable cohort studies by the combination of appropriate pulse sequences and cryogenically cooled resonators (CCR). The objective of this study was to apply this DTI approach at the group level to β-amyloid precursor protein (APP) transgenic mice.

**Methods:**

Twelve mice (5 wild type, 7 APP transgenic tg2576) underwent DTI examination at 156^2^×250 µm^3^ spatial resolution with a CCR at ultrahigh field (11.7 T). Diffusion images were acquired along 30 gradient directions plus 5 references without diffusion encoding with a total acquisition time of 35 minutes. Fractional anisotropy (FA) maps were statistically compared by whole brain-based spatial statistics (WBSS) at the group level vs. wild type controls.

**Results:**

FA-map comparison showed characteristic regional patterns of differences between the groups with localizations associated with Alzheimer’s disease in humans, such as the hippocampus, the entorhinal cortex, and the caudoputamen.

**Conclusion:**

In this proof-of-principle study, regions associated with amyloid-β deposition could be identified by WBSS of FA maps in APP transgenic mice vs. wild type mice. Thus, DTI in the mouse brain acquired at 11.7 T by use of a CCR was demonstrated to be feasible for cohort studies.

## Introduction

Noninvasive imaging of brain pathology associated with Alzheimer’s disease (ALZD) is mandatory for further understanding of the pathophysiology as well as for its diagnosis and for treatment selection and monitoring [1]. The Alzheimer’s Disease Neuroimaging Initiative was founded in order to assess ALZD by various imaging biomarkers [2], and magnetic resonance imaging (MRI)-based biomarkers proved high potential for the early detection and monitoring of ALZD [3]–[5]. Recently, diffusion tensor imaging (DTI) has been promisingly applied as an imaging correlate to the assessment of human ALZD [6], [7].

As the most common type of dementia, ALZD is characterized by a progressive loss of neuronal function, leading to gradual memory impairment, confusion, and general loss of social functioning [2], [8]. Pathologic hallmarks are the accumulation of extracellular amyloid plaques, caused by amyloid-b protein aggregation, and the presence of intracellular neurofibrillary tangles, formed by aggregates of the hyperphosphorylated tau protein [8]. These pathological changes originate in the medial temporal lobe, especially the entorhinal cortex and hippocampus, spreading further across the limbic cortex and neocortex [8], [9].

For preclinical research, there has been increasing effort to develop similar MRI-based biomarkers of the pathological changes in animal models of ALZD *in vivo* [10], [11] as well as *ex vivo* [1]. In this context, DTI has also evolved as an increasingly important tool for studying the anatomy of the mouse brain both *in vivo* [12], [13] and *ex vivo* [14]. In a recent study [15], the feasibility of rapid *in vivo* fiber tracking and microstructural analysis of the mouse brain was demonstrated utilizing the signal-to-noise ratio (SNR) gain of a cryogenic cooled resonator (CCR). The resulting acquisition time of 35 minutes proven in this study enabled large scale *in vivo* whole brain murine DTI cohort studies. In contrast to previous studies [8], [16], [17] where group comparisons had been performed at subject coordinate space level level (e.g. by individual ROI analyses), in the current study group comparison was performed after stereotaxic normalization on a study-specific template on a voxelwise basis. In the context of DTI research, the objective of this study was to show the feasibility of rapid DTI measurements in cohort studies in APP transgenic mice (APP mice) vs. wild type mice (wt mice) for the identification of diffusion related brain differences. Rapid acquisition was realized by combining ultrahigh field MRI and cryo-cooled probe. The presented work aims as the basis for future studies aiming at examining experimental model of ALZD and other neurodegenerative diseases.

.

## Results

A summary of regional FA alterations including cluster sizes are listed in **Table 1**. Significant differences in the DTI-based fractional anisotropy (FA) between APP and wt mice were observed. Whole brain-based spatial statistics (WBSS) showed a widespread characteristic and symmetric pattern of FA differences between the two cohorts. Cluster locations of FA reduction (i.e., APP< wt) were observed in the caudoputamen (left and right hemisphere), the caudoputamen near the ventral hippocampus (left and right hemisphere), the dorsal hippocampus (left and right hemisphere), the entorhinal cortex (left and right hemisphere), and the thalamus. Further areas of regional FA reduction were observed in the internal capsules (left and right hemisphere), clusters in the paramedian raphe nucleus area and in the periaqueductal grey.

**Table 1:** Resulting clusters obtained by whole brain-based spatial statistics (WBSS) of FA maps from APP mice compared to wt mice (cluster size in pixels).

	anatomical localization	hemisphere	cluster size
**FA reduction**	caudoputamen	R	74
		L	550
	caudoputamen/ventral hippocampus	R	85
		L	360
	dorsal hippocampus	R	483
		L	166
	entorhinal cortex	R	1860
		L	1027
	thalamus	median	559
	internal capsule	R	238
		L	198
	paramedian raphe nucleus area	median	2038
	periaqueductal grey/dorsal raphe nucleus	median	950
**FA increase**	lateral septal nucleus/ventral ventricles area	median	2537
	lateral cerebellum	R	210
		L	938

**Figure 1:**
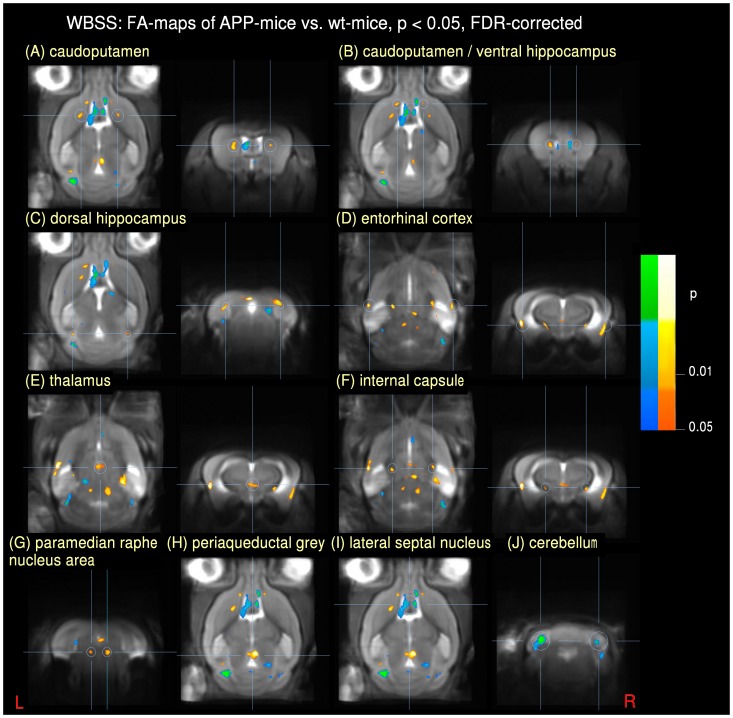
Results from whole brain-based spatial statistics (WBSS) of FA-maps of APP mice vs. wt mice at p<0.05, FDR corrected (axial and/or coronal views). Increase is displayed in cold colors, reduction is displayed in hot colors. (A) bihemispheric reduction in the caudoputamen; (B) bihemispheric reduction in the caudoputamen/ventral hippocampus; (C) bihemispheric reduction in the dorsal hippocampus; (D) bihemispheric reduction in the entorhinal cortex; (E) reduction in the thalamus; (F) bihemispheric reduction in the internal capsule; (G) reduction in the paramedian raphe nucleus area; (H) reduction in the periaqueductal grey/dorsal raphe nucleus; (I) increase in the lateral septal nucleus area; (J) bihemispheric increase in the cerebellum.

Clusters of FA increase (i.e., APP> wt) were located near the lateral septal nucleus area and the ventral ventricles and in the lateral cerebellum (left and right hemisphere). **Figure 1** gives an overview on the symmetrical distribution of APP-related FA clusters.

**Figure 2:**
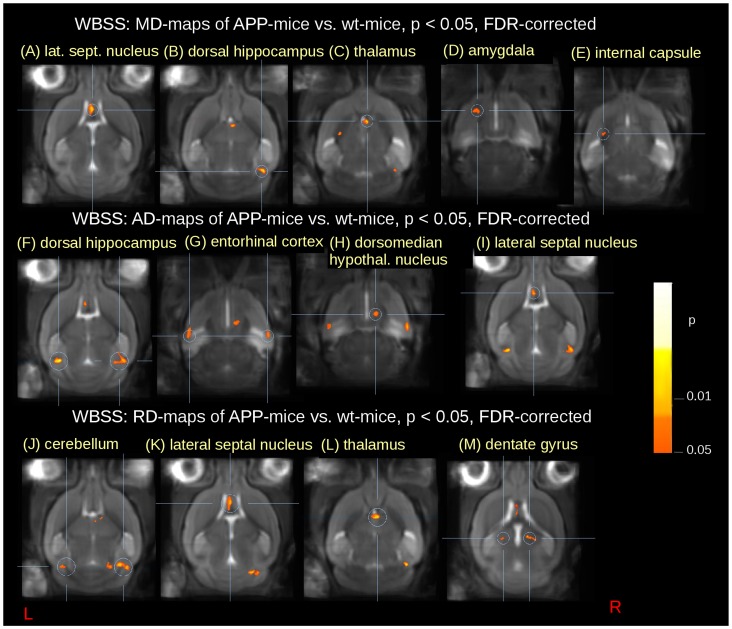
Results from whole brain-based spatial statistics (WBSS) of MD-, AD-, and RD-maps of APP mice vs. wt mice at p<0.05, FDR corrected (coronal views). Reduction is displayed in hot colors. (A–E) MD reduction in the lateral septal nucleus, the dorsal hippocampues, the thalamus, the amygdala, and the internal capsule, respectively; (F–I) AD-reduction in the dorsal hippocampus (bihemispheric), the entorhinal cortex (bihemispheric), the dorsomedian hypothalamic nucleus region, and in the lateral septal nucleus, respectively; (J–M) RD-reduction in the cerebellum (bihemispheric), the lateral septal nucleus, the thalamus, and the dentate gyrus, respectively.

Other DTI metrics also showed differences in comparison APP mice vs. wt mice: Significant reduction in mean diffusivity (MD) between APP and wt mice (**Figure 2A–E** **,** **Table 2**) indicating a regional loss of diffusivity observed in the lateral septal nucleus area (corresponding to FA increase), in the dorsal hippocampus, and small clusters of reduction in the amygdala and in the internal capsule.

**Table 2:** Resulting clusters obtained by whole brain-based spatial statistics (WBSS) of MD, RD, and AD maps from APP mice compared to wt mice (cluster size in pixels).

	anatomical localization	hemisphere	cluster size
**MD-reduction**	lateral septal nucleus/ventral ventricles area	median	1704
	dorsal hippocampus	R	1598
	thalamus	median	765
	amygdala	L	519
	internal capsule	L	312
**AD-reduction**	dorsal hippocampus	R	1919
		L	1242
	entorhinal cortex	L	1087
		R	1258
	dorsomedian hypothalamus nucleus region	R	335
	lateral septal nucleus	median	253
**RD-reduction**	lateral cerebellum	L	212
		R	1985
	lateral septal nucleus	median	1410
	thalamus	median	573
	dentate gyrus	R	151
		L	98

Decreases in FA do not distinguish between myelin degeneration and axonal injury. Therefore, in addition to FA and MD analysis, calculation of axial diffusivity (AD) which is related to axonal injury, and radial diffusivity (RD) which represents myelin degeneration may complement the results [18]. AD reductions (**Figure 2F–I** **,** **Table 2**) were observed bilaterally in the dorsal hippocampus and in the entorhinal cortex underlining the FA reduction in the corresponding regions, thus indicating axonal injury. Further smaller clusters of AD reduction were observed in the dorsomedial hypothalamic nucleus and in the lateral septal nucleus.

RD reductions (**Figure 2J–M** **,** **Table 2**) were observed bilaterally in the cerebellum (corresponding to FA increase) and bilaterally in the dentate gyrus (indicating myelin degeneration). Further clusters could be found in the lateral septal nucleus and in the thalamus region.

Due to the template specific normalisation, the coordinates likely differ from coordinates in Paxinos and Franklin [19]. Thus, locations of clusters are not presented in Paxinos’ coordinate frame.

Data analysis was performed at an individual voxel level in stereotaxic coordinate space. This analysis in stereotaxic space may reduce the sensitivity of imaging metrics to detect pathology. Therefore, data from ROIs (bilateral thalamus and bilateral entorhinal cortex) in individual-mouse space were compared to clusters in the equivalent anatomical location in stereotaxic space. The percentage of differences between the means of such data and their p-value differences were listed in **Table 3**. The WBSS analysis in stereotaxic space revealed similar results compared to ROI analysis in individual space.

**Table 3:** Differences in significance comparing native space ROI-analysis and stereotaxic space ROI-analysis (APP mice vs. wt mice).

group differences APP vs. wt – ROI (15 mm)						
		thalamus		entorhinal cortex		
**mean FA values**						
	APP		wt		APP	wt
native space	0.25		0.32		0.30	0.37
stereotaxic space	0.29		0.34		0.30	0.36
**p-values**						
native space	p = 0.003				p = 0.015	
stereotaxic space	p = 0.032				p = 0.001	

Bihemispherical data were arithmetically averaged.

## Discussion

Previous DTI studies of APP mice revealed widespread patterns associated with amyloid deposition: Song et al. [17] related amyloid deposition and FA reductions to regions such as cerebral peduncle, corpus callosum, external capsule, and fornix (see also [16]). Further ROI-based FA studies focussed on the corpus callosum and the ventral hippocampal commissure [1]. A more recent study [8] reported (besides other DTI metrices such as MD, AD, and RD) FA reduction in the splenium of the corpus callosum, the fimbria and in the cortex, whereas FA increase was found in the cerebral peduncle, in the internal capsule, and in the lateral posterior thalamus nuclei.

DTI studies of ALZD in humans reported altered FA in multiple areas [7], and despite somehow controversial reports, there are three regions that present consistent findings of reduced FA. These regions are consistent with the development of ALZD-associated pathological changes and include (i) sub-regions of the medial temporal lobe including the hippocampus, entorhinal cortex and parahippocampal white matter, (ii) temporal lobes, and (iii) the posterior cingulum – for a review see [7].

As multiparametric results from this study bilateral DTI metric alterations in the dorsal hippocampus and in the entorhinal cortex suggest axonal injury. Bilateral FA reductions in the caudoputamen and internal capsule could not be related to either axonal injury or myelin degeneration. FA increase in the cerebellum combined with RD decrease suggests myelin degeneration in not coherently aligned fiber structures. Small bilateral RD decrease in dentate gyrus should be topic to further research.

Although, a direct fit of the results from mice to humans could not be drawn, parallels and analogies should be discussed: The regions identified by FA alterations at the group level in this animal study were mainly related to anatomical structures that were already identified to be ALZD related in human DTI studies [7], i.e. hippocampus and entorhinal cortex. The thalamus, the internal capsule and the hippocampus have also been identified by DTI metrics comparing APP and wt mice [8]. Additionally, regions that are prone to be associated with ALZD were identified, e.g. the caudoputamen, as well as the thalamus [2]. Further regions that were identified for FA reduction were in the paramedian raphe nucleus region and in the periaqueductal grey near the dorsal raphe nucleus. Involvement of the raphe nuclei in human ALZD has already been reported [20].

FA reductions (wt>APP) changes may be indicative of axonal degeneration (i.e., reduced water diffusion parallel to axonal tracts) and myelin degradation (i.e., increased water diffusion perpendicular to axonal tracts), whereas FA increases (APP> wt) could appear in regions of crossing fibers or in nuclei with loss of axonal structures in one direction (i.e. the remaining directionality is mapped) [21]. The FA increase in the lateral septal nuclei may be related to the complex nuclei structure or could be related to residuals of the stereotaxic normalization of the ventral ventricles which could be increased during the development of APP mice as a result of a global brain atrophy [22].

There are limitations to the present study. As the number of animals in each group was small, additional clusters showing altered FA values which could not be associated with APP-related changes likely appear in the sense of false positives. However, the hemispherical symmetry of the alterations in areas associated with APP may be considered an indicator of reliability. The cluster sizes of caudoputamen and caudoputamen/ventral hippocampus in the right hemisphere differ from their left counterparts. We suggest that by comparing these FA results with histological findings at a later time the reasons behind these left-right asymmetries may be established.

Data from a histological evaluation were currently not available. We hope to provide histological data at a later time to correlate DTI findings with histology especially to address the question of amyloid*-β* plaque load, i.e. all locations of significantly altered FA should be correlated with histological findings at a later time to ascertain whether or not these are truly false positives.

Neuropil threads, pretangles, and neurofibrillary tangles are hallmark tau lesions of AD. However, inasmuch as neurofibrillary changes are found only in mouse lines that overexpress mutated tau or human tau on a murine tau −/− background, we are unable to comment on the role of tau here because the model available to us was not designed to express human tau pathology.

The mice in this study were comparatively old (23 months). Nevertheless, in order to show the methodological applicability, the selected mouse cohort seemed to be appropriate. In order to address axonal changes and myelin degeneration, application to a younger mouse cohort should be performed. This is a task for a future study.

As a methodological limitation, the fast DTI imaging with one signal accumulation applied in this study has the potential drawback of motion corrupted volumes. A specific technique for the detection and elimination of such corrupted volumes has been applied [23]. In detail, in ten data sets less than 5 gradient directions had to be eliminated and in two data sets 10 gradient directions were eliminated. Furthermore, we did not perform *ex vivo* histological examinations in this study to complement the *in vivo* imaging findings [24].

Cardiac gating and especially respiratory gating can be applied for further improving image fidelity. Nevertheless, in this specific protocol only slight improvement in the overall image quality was observed, thereby substantially sacrificing image acquisition time, a quality check was applied and corrupted volumes were removed before data analysis. In the presented study, in none of the animals more than 5 volumes had to be extracted and rescanning was not required in any case.

In order to calculate differences between hypothesis-driven ROI analysis in native space and hypothesis-free WBSS in stereotaxic space, exemplarily results of regions with FA-decrease were compared. The results of ROI-analysis results in individual mouse space and cluster-values in stereotaxic space were in accordance.

This study demonstrated the feasibility of cohort DTI studies in a mouse model of ALZD. The rapid imaging protocol at 11.7T [15] enabled sufficient image quality for subsequent analysis at the group level by WBSS. Significant differences in the FA values between wt and APP mice could already be observed in small animal cohorts.

.

## Materials and Methods

### 1. Animals

All experiments were performed in accordance with German animal protection laws and had been approved by the national animal board (VVH 11/009, Regierungspräsidium Baden-Württemberg, Tübingen, Germany).

Seven mice (tg2576) of APP transgenic type C57BL6/SJL [25] (23 months old) and corresponding five adult wild type mice C57BL/6 (23 months old) underwent whole-brain DTI-MRI. All data acquisition was carried out under isoflurane anesthesia (3% for induction and 1.5% for maintenance). The animals were placed in a stereotaxic head support (Bruker Biospin, Ettlingen, Germany) to immobilize the head. Body temperature was maintained by an integrated water based heating device. The actual body temperature of the mouse was monitored by a rectal temperature probe and respiration was monitored by an air-filled pillow positioned under the abdomen of the mouse. The breathing frequency was maintained at 75–80 cycles per minute. The mice rapidly recovered after the termination of anesthesia at the end of the MRI procedure.

### 2. Data Acquisition

Imaging was performed with an 11.7 T small bore animal scanner (Biospec 117/16, Bruker, Ettlingen, Germany), BGA-9S gradient system with a maximum gradient strength of 760 mT/m and a slew of 6840 T/m/s. A two-element transmit/receive ^1^H mouse cryogenic surface coil (CryoProbe, Bruker BioSpin) was used for data acquisition. Imaging parameters of the optimized rapid diffusion prepared segmented spin echo EPI imaging protocol were as: TE/TR 50.5 ms/15000 ms, bandwidth 499 kHz, matrix 128×96, in-plane resolution 156 µm×156 µm, 60 axial slices with a slice thickness of 250 µm, four segments. In order to transfer analysis procedures proven valuable in human DTI similar volume to voxel size relations as in humans were used for the mouse scans. The required voxel resolution and brain grid coverage resulted: in-plane (sagittal, coronar) brain grid of approximately 50 voxels, and an axial grid of approximately 20 voxels to match comparative range as brain grid coverage from standard established human DTI. Thirty diffusion directions (gradient duration 4 ms, spacing 10 ms, and amplitude 345 mT/m) with b = 1000 s/mm^2^ and five unweighted b = 0 volumes (standard gradient scheme as provided by the Bruker software), one signal average, were acquired, resulting in a total acquisition time of 35 minutes [15]. No respiratory or cardiac synchronization was used.

### 3. Data Analysis/Processing

**Figure 3:**
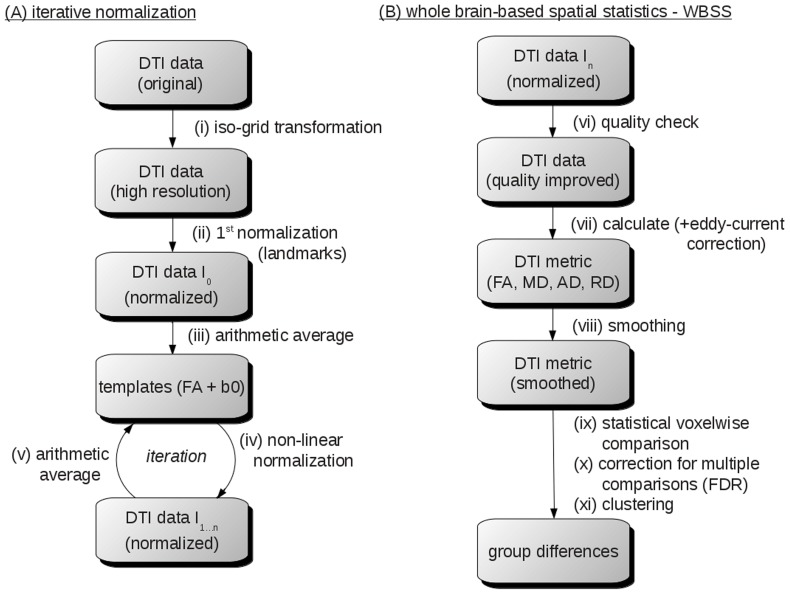
Workflow of the iterative normalization process and the whole brain-based spatial statistics. (**A**) (i) recorded data were transformed into a 50 µm isogrid (nearest neighbor interpolation). (ii) after linear transformation according to manually set landmarks identified using a stereotaxic mouse atlas (iii) scanner- and sequence specific b0- and FA-templates were created. (iv) data were non-linearily normalized and templates were created (v) in an iterative process. (**B**) whole brain-based spatial statistics (WBSS) was performed after a quality check (vi) to eliminate motion corrupted volumes. After eddy-current correction, DTI-metrics were calculated (vii). Smoothing of the DTI-metrics maps (viii), statistical voxelwise comparison (ix), correction for multiple comparisons (x), and clustering (xi) lead to significant group differences.

Recorded data were transformed into a 50 µm isogrid (nearest neighbor interpolation) in order to minimize partial volume effects. The slice thickness to in-plane resolution ratio of 1.6 as well as recorded brain grid was in the same order as in human DTI studies (see e.g. [26], [27]). The transformation to an iso-grid of 50 µm corresponds to an iso-grid of 1 mm in human studies. As brain coverage grid and slice thickness to in-plane resolution ratio were similar to human DTI studies, data processing was performed with the *Tensor Imaging and Fiber Tracking* (TIFT) software package [28] which has previously been successfully applied to human DTI group studies (e.g. [29]). A flow chart of the whole data processing procedure (stereotaxic normalisation and WBSS) is provided in **Figure 3**.

Spatial normalization to a stereotaxic standard space was performed using a study-specific b0-template and an FA-template (see flow chart provided in **Figure 3A**). Normalization to an FA-template was included into the process since a non-linear registration to an FA-template has the advantage that it provides more contrast in comparison to b0-images [30].

The whole normalization process was iterative. Scanner- and sequence specific b0- and FA-templates were created in a first step by arithmetically averaging data sets of all mice after linear transformation according to manually set landmarks identified using a stereotaxic mouse atlas [19]. After this first iteration, data were non-linearily normalized during a second iteration step in order to further optimize the normalization matrices. This process was iteratively repeated until correlation between individual FA-maps and the FA-template was >0.7, which was usually achieved after two iterations.

The flow chart of the whole brain-based spatial statistics was provided in **Figure 3B**. Motion artefacts were eliminated in each data set and each mouse separately by a recently described quality check procedure [23]. After eddy-current correction, DTI metrics were calculated according to standard methods [21]:

The second-rank diffusion tensor 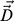 can always be diagonalized leaving only three non-zero elements along the main diagonal of the tensor, i.e. the Eigenvalues 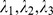. The Eigenvalues reflect the shape or configuration of the ellipsoid. The mathematical relationship between the principal coordinates of the ellipsoid and the laboratory frame is described by the Eigenvectors 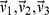. Since there are several challenges in displaying tensor data, the concept of diffusion ellipsoids has been proposed [31]. The Eigendiffusivities of these ellipsoids represent the unidimensional diffusion coefficients in the main direction of diffusivities of the medium, i.e. the main axis of the ellipsoid represents the main diffusion direction in the voxel which coincides with the direction of the fibers, while the eccentricity of the ellipsoid provides information about the degree of anisotropy and its symmetry. Therefore, diffusion anisotropy metrics can be defined [32], [21].

### Mean Diffusivity (MD)



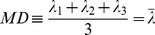
(1)Radial Diffusivity (RD)
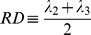
(2)


### Axial Diffusivity (AD)

### Fractional Anisotropy (FA)



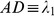
(3)

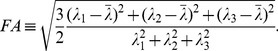
(4)Since filter size influences results of the DTI data analysis [33], adjustment of the smoothing kernel was done according to the matched filter theorem, which states that the width of the filter used to process the data should be tailored to the size of the expected difference. Accordingly, an 8 voxel (400 µm) full width at half maximum (FWHM) Gaussian filter was applied for filtering to balance the trade-off between sensitivity and specificity (see also e.g. [26]). Voxelwise statistical comparison between FA maps of APP mice and wt mice was performed by Students t-test to infer significant differences between both groups. Correction for multiple comparisons by the false-discovery-rate (FDR) algorithm at a nominal level of p<0.05 was used [34]. Further reduction of the alpha error was achieved by a spatial correlation algorithm that eliminated isolated voxels or small isolated groups of voxels, leading to a threshold cluster size of 64 contiguous significant voxels.
